# Dynamic Changes of Inhibitory Killer-Immunoglobulin-Like Receptors on NK Cells after Allogeneic Hematopoietic Stem Cell Transplantation: An Initial Study

**DOI:** 10.3390/jcm9113502

**Published:** 2020-10-29

**Authors:** Tereza Dekojová, Lucie Houdová, Jiří Fatka, Pavel Pitule, Pavel Ostašov, Valentina S. Caputo, Hana Gmucová, Daniel Lysák, Pavel Jindra, Monika Holubová

**Affiliations:** 1 Department of Haematology and Oncology, University Hospital Pilsen, 304 60 Pilsen, Czech Republic; DEKOJOVAT@fnplzen.cz (T.D.); Pavel.Pitule@lfp.cuni.cz (P.P.); GMUCOVAH@fnplzen.cz (H.G.); LYSAK@fnplzen.cz (D.L.); JINDRA@fnplzen.cz (P.J.); 2 Department of Histology and Embryology, Faculty of Medicine in Pilsen, Charles University, 301 66 Pilsen, Czech Republic; 3 NTIS, Faculty of Applied Science, University of West Bohemia, 301 00 Pilsen, Czech Republic; houdina@kky.zcu.cz (L.H.); fatkaj@ntis.zcu.cz (J.F.); 4 Laboratory of Tumor Biology and Immunotherapy, Biomedical Center, Faculty of Medicine in Pilsen, Charles University, 323 00 Pilsen, Czech Republic; Pavel.Ostasov@lfp.cuni.cz; 5 Department of Immunology and Inflammation, Faculty of Medicine, Imperial College London, London W12 0NN, UK; v.caputo@imperial.ac.uk

**Keywords:** KIRs, NK cells, HSCT

## Abstract

Killer-immunoglobulin-like receptors (KIRs) are critical natural killer (NK) cell regulators. The expression of KIRs is a dynamic process influenced by many factors. Their ligands—HLA(Human Leukocyte Antigen) class I molecules—are expressed on all nucleated cells that keep NK cells under control. In hematopoietic stem cell transplantation (HSCT), NK cells play an essential role in relapse protection. In the presented pilot study, we characterized the dynamic expression of inhibitory KIRS (iKIRs), which protect cells against untoward lysis, in donors and patients during the first three months after HSCT using flow cytometry. The expression of all iKIRs was highly variable and sometimes correlated with patients’ clinical presentation and therapy regiment. Cyclophosphamide (Cy) in the graft-versus-host disease (GvHD) prevention protocol downregulated KIR2DL1 to just 25% of the original donor value, and the FEAM (Fludarabine + Etoposid + Ara-C + Melphalan) conditioning protocol reduced KIR2DL3. In lymphoid neoplasms, there was a slightly increased KIR2DL3 expression compared to myeloid malignancies. Additionally, we showed that the ex vivo activation of NK cells did not alter the level of iKIRs. Our study shows the influence of pre- and post-transplantation protocols on iKIR expression on the surface of NK cells and the importance of monitoring their cell surface.

## 1. Introduction

Allogeneic hematopoietic stem cell transplantation (HSCT) represents an essential treatment in many types of high-risk hematological malignancies, such as acute leukemias, myelodysplastic syndrome (MDS), and relapsing/refractory lymphomas. The patients’ hematopoiesis and immune system are first destroyed with a preparative regimen, previous to HSCT. After infusion of the donor’s hematopoietic stem cells (HSC) and their engraftment, a new immune system develops and protects patients against cancer recurrence mediated by the graft-versus-tumor (GvT) effect. Natural killer (NK) cells play a crucial role in the GvT effect and also provide significant antiviral protection (i.e., Cytomegalovirus (CMV)). Moreover, they can also favor engraftment by killing lymphohematopoietic host cells and suppressing graft-versus-host disease (GvHD) by eliminating host dendritic cells (DCs) [1,2]. NK cells are the first lymphoid lineage cell type reconstituted after allo-HSCT and remain the dominant circulating lymphocyte population during the first three months post-transplant [3]. However, CMV infection, corticosteroids, or the presence of GvHD can affect the reconstitution of NK cells [4,5].

**Figure 1:**
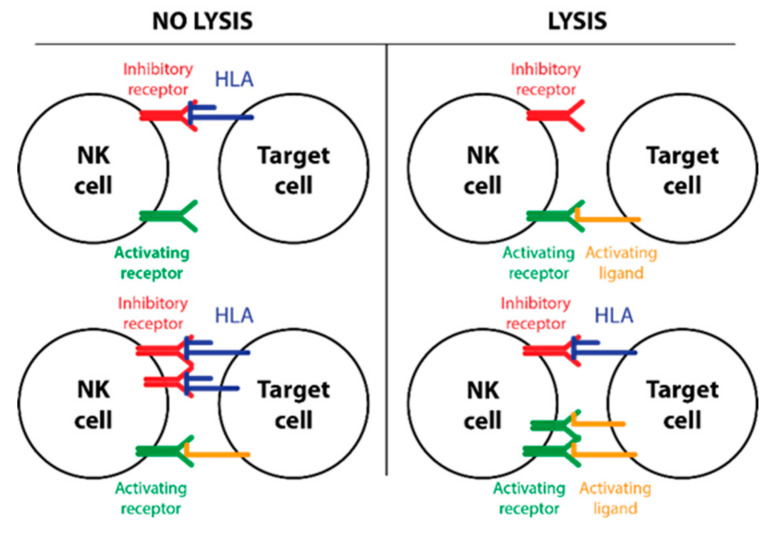
The regulation of natural killer (NK) cell activity is based on the balance between activating and inhibitory signals. NK cells are muted in cases of the absence of an activating signal or stronger inhibitory signals compared with activating ones. The NK cells are fully activated and kill the target cell when the ligand for inhibitory signals is missing or when the activating signals exceed inhibitory ones.

NK cells’ immune response is determined by a balance between signals received from inhibitory and activating receptors (Figure 1). There are three main types of NK cell receptor families: Activating and inhibitory killer-immunoglobulin-like receptors (KIRs) (interacting with HLA class I); activating and inhibitory C-type lectin receptors (interacting with non-HLA molecules MIC-A, MIC-B, UL16 binding protein family, and HLA-E); and activating natural cytotoxicity receptors (NCR) with a high variability of ligands [6,7]. KIR receptors have a prominent role in NK cell regulation and are recognized as critical non-HLA factors affecting the outcome of allogeneic stem cell transplantation and are widely used during the selection process of the best donor for hematopoietic stem cell transplantation. KIR receptors are encoded by 16 KIR genes. These receptors can contain three (KIR3D) or two (KIR2D) domains in the extracellular region. The domains feature either a long “L” (inhibitory KIRs) or short “S” (activating KIRs) cytoplasmic tail (intracellular domain) [8,9]. The KIR gene content is heterogeneous among human individuals, and there are almost 600 genotypes in the world (data obtained from www.allelefrequencies.net). The most direct classification of KIR genotypes divides them into two major haplotypes—A and B. Group B haplotypes are characterized by one or more of the following genes: *KIR2DL5*; *KIR2DS1*; *KIR2DS2*; *KIR2DS3*; *KIR2DS5*; and *KIR3DS1*. Group A haplotypes are determined by the absence of all these genes and the presence of only one activating gene (*KIR2DS4*) [10]. Simply, the haplotype A group contains inhibitory receptors, and the haplotype B group includes a combination of activating and inhibitory ones. The B haplotype and its composition are associated with a lower risk of relapse [11].

The level of KIRs on the NK cell surface can be crucial for controlling malignant disease. The expression level depends on the presence of an HLA class I molecule or the number of KIR genes [12], and it is determined during a process called the education of NK cells, essential for protecting healthy cells from lysis (setting of the optimal negative signal).

There are two types of KIR–HLA interactions. The straightforward reaction is an immune response where HLA ligands interact with the inhibitory or activating KIRs, leading to either the suppression or activation of NK cells. HLA class I molecules are downregulated on infected cells or tumor cells, causing an elevation of activating signals as a result of the absence of inhibitory signals [13]. This is known as “missing-self”. The “missing-self” interaction is the basis of HSCT anti-cancer capabilities in the case where donors’ KIR receptors do not have corresponding HLA ligands on patients’ cells (KIR–HLA mismatch) [14]. This situation leads to a potential decrease in the inhibition and promotion of the anti-cancer response. The non-direct KIR–HLA interaction is meaningful during NK cell development. The education and licensing are processes of NK cell maturation in which NK cells are tested in terms of their responsiveness, and the balance of inhibitory/activating signals is screened and tuned [15].

Currently, NK cells are being used in cellular therapy for the prevention or treatment of relapse after allo-HSCT. They are mostly ex vivo activated by several protocols, where the presence of cytokines or feeder cells can influence the KIR expression [16]. The primary sources employed for ex vivo NK cell preparation are healthy donors, but the limited number of donors negatively affects the potential of NK cell applications. There are also off-the-shelf NK cells which can potentially be applied to any patients, but with the risk of immune-related rejection [17]. Using NK cells from patients after allogeneic HSCT represents an important source for almost all transplanted patients, but there are many fundamental questions, including those relating to the timing of collection. Although NK cell reconstitution is observed around one month post-transplant, it takes several months to gain immunophenotypic and functional characteristics comparable to healthy donors [18]. The condition of transplanted patients is unpredictable, and it is impossible to guarantee sufficient NK cell numbers with an appropriate quality several months after HSCT because of the prolonged immunosuppression and GvHD. Because the crucial factor in NK cell reactivity is excess of the inhibition signal, we monitored the inhibitory KIR receptor expression on the surface of NK cells in donors before HSC collection and at early stages (1, 2, and 3 months) after HSC transplantation in patients, in order to see what would be the best time window for NK cell collection (i.e., the lowest expression of inhibitory KIRs-inhibitory KIRS (iKIRs)). Moreover, we evaluated the influence of our clinical-grade culture protocol on the level of inhibitory KIRs.

## 2. Methods

### 2.1. Donor/Patient Samples and Data

The ethics committee approved the study (joint committee of the Faculty of Medicine in Pilsen and Faculty Hospital Pilsen) on 4 September 2014. Signed informed consent was obtained from all participants enrolled in the study. Peripheral blood of allogeneic donors was collected during their medical evaluation (work-up) about 1 month before a collection of HSC, together with the infection disease status. Blood samples from patients were collected 1, 2, and 3 months after HSCT, along with an evaluation of the clinical data (diagnosis, GvHD status, disease status, CMV infection presence and treatment, cyclosporin A level, and corticosteroid/Kg). To evaluate the role of ex vivo activation on iKIR expression, mononuclear cells from six different patients for further NK cell isolation were cryopreserved at the second time point. The cohort’s specifications are shown in Table 1.

### 2.2. NK Cell Isolation and Culture

To test the influence of ex vivo activation on iKIR expression, the NK cells were isolated from six patients two months after transplantation and cultured for approximately 3 weeks in concordance with the clinical-based protocol used in the clinical trial EudraCT number: 2018-001562-42. NK cells were isolated from mononuclear cells (MNCs) using an NK cell isolation kit (Miltenyi, Bergisch Gladbach, Germany), following the manufacturer’s instructions. Isolated NK cells were seeded with feeder cells (pooled allogeneic MNCs from five donors irradiated by 25 Gy) at a ratio of 1:10 (NK:feeder cells) and cultured in SCGM medium containing 5% AB plasma (local source) and IL-2 (1000 IU, Proleukin, Novartis, Basel, Switzerland). IL-2 was added every 2–3 days. Cells were passaged after 10 days and at the end of the culture period using Tryple Select reagent (Gibco, Grand Island, NY, USA) for the de-attachment of adherent cells. The purity and iKIR expression were measured at the end of the experiment (18 days after isolation).

To test the role of the KIR2DL1 receptor in the inhibition of NK cells, we blocked the HLA-KIR2DL1 signal by employing an antibody against the KIR2DL1 receptor and co-cultured NK cells from four different donors with KIR2DL1 expression against the NK-resistant KG1a AML cell line (Sigma Aldrich, St. Louis, MO, USA) carrying a ligand for this receptor. NK cells were cultured with the presence of anti-KIR2DL1 antibody (Miltenyi, Bergisch Gladbach, Germany) for 2 h, followed by the addition of KG1a at a ratio of 10:1 (NK:KG1a). The cytotoxic activity against KG1a in KIR-HLA blocked or unblocked cultures was compared. The experiment was repeated twice for each donor.

### 2.3. Flow Cytometry

The expression of iKIR receptors was measured by flow cytometry. EDTA-treated peripheral blood (100 µL) or a cell suspension was stained with a mixture of antibodies (backbone markers) CD45-HorV500 (BD Bioscience, San Jose, CA, USA), CD3-PB (Exbio, Vestec. Czech Republic), CD56-PE-Vio770 (Miltenyi Biotec, Bergisch Gladbach, Germany), and CD16-PerCP (Exbio, Vestec. Czech Republic) combined with KIR2DL1-PE, KIR2DL2/2DL3-APC, KIR2DL3-FITC or KIR3DL1/3DL2-PE, KIR2DL5-APC, and KIR3DL1-FITC (all KIR antibodies—Miltenyi Biotec, Bergisch Gladbach, Germany) for 10 min at 4 °C. After incubation, lysing solution (1x ammonium chloride; red blood cell lysis) was added for 10 min at room temperature (RT), followed by washing with PBS and centrifugation at 350 g/5 min/RT. The cultured cells were washed, omitting the lysing step.

To detect the KG1a cell line in co-culture experiments (see Section 2.2), we used the CD34-PC7 antibody (Beckman Coulter, Brea, CA, USA) and 7AAD (Exbio) for the cell viability evaluation. The cell suspension was stained with the antibody mixture for 15 min at RT, followed by washing with PBS for 5 min/300 g/RT. 

After the washing step (in both protocols), the cell pellets were resuspended in 300 uL of PBS, and samples were immediately measured on a BD FACSCanto II flow cytometer (BD). The application settings using actual CST values (measured using CS&T beads—Cytometer Set-Up and Tracking beads) were used for the standardization of day-to-day variability. The analysis was conducted using FlowJo software (FlowJo LLC, BD Bioscience, San Jose, CA USA). The complete gating strategies are shown in Supplementary Materials, Figure S1A–D.

### 2.4. KIR Genotyping

DNA from both patients’ and donors’ whole blood was isolated using the Maxwell 16 Blood DNA purification kit and Maxwell^®^ 16 Instrument (both Promega, Madison, WI, USA). The DNA concentration and purity were determined by spectrophotometric determination at A260; purity was determined based on the A260/A280 ratio (Synergy HTX instrument–BioTek, USA).

The complete KIR genome was amplified by the Long-Range (LR) PCR protocol modified from Maniangou et al., 2017 [17]. A mixture of four forward (5′-GCCAAATAACATCCTGTGCGCTGCTGAGCT-3′, 5′-CTCACAACATCCTGTGTGCTGCTGAACTGA-3′, 5′-GCGGCCGCCTGTCTGCACAGACAGCACC-3′, and 5′-CACATCCTCTGCACCGGTCAGTCGAGCCGA-3′) and two reverse (5′-TTGGAGAGGTGGGCAGGGGTCAAGTG-3′ and 5′-CTCCATCTGAGGCTCCCCTGAATGTG-3′) primers was used in one multiplexed LR-PCR at a final concentration of 0.2 uM of each primer, with 2 uL of PrimeSTAR GXL DNA Polymerase, 1× PrimeSTAR GXL Buffer, and dNTP mixture, forming samples of 200 µM (all from Takara Saint-Germain-en-Laye, France). Then, 50 uL PCR reactions were performed in a CFX96 PCR cycler (Bio-Rad, USA) with an initial denaturation step of 30 s at 98 °C, followed by 31 cycles of 10 s at 98 °C, 30 s at 68 °C, and 12 min at 72 °C, and a final elongation step for 10 min at 72 °C. PCR products were analyzed by agarose gel electrophoresis and visualized by SYBR Safe DNA Gel Stain (Thermo Scientific, Waltham, MA, USA), and the sizes of products were compared with the GeneRuler 1 kb Plus DNA Ladder (Thermo Scientific). The Qubit™ dsDNA High Sensitivity Assay Kit (Thermo Scientific) was used to determine the PCR product concentration.

Sequencing libraries were prepared from 250 or 100 ng of input amplified genomic DNA using the NEBNext Ultra II FS DNA Library Prep Kit for Illumina, and barcoded with Multiplex Oligos for Illumina (both New England Biolabs, Ipswich, MA, USA). Library preparation followed the manufacturer’s protocol, but all steps were performed with half of the recommended volumes. Agencourt AMPure XP beads (Beckman Coulter, Brea, CA, USA) were used for selection of the optimal library size. Library quality control was performed by the Qubit™ dsDNA High Sensitivity Assay Kit (Thermo Scientific) for the concentration and High Sensitivity DNA ScreenTape in combination with Agilent 2200 TapeStation (Agilent, Santa Clara, CA, USA) for library size determination. Barcoded libraries that passed quality control were pooled and sequenced on the MiSeq instrument (Illumina, San Diego, CA, USA) to produce 2 × 251 bp paired-end reads.

KIR allele identification was performed by using an in-house bioinformatic pipeline designed as a knowledge-based iterative process for result space reduction using parameters such as the allele acceptance function for each KIR gene, primarily determined by the depth of coverage and allele coverage, resulting in the allele score, and the decision function for continuation or termination of iteration, using available knowledge from the IPD-KIR database (release 2.9.0) and information from previous studies [19,20]. The software is available on http://www.kky.zcu.cz/en/sw/KIRlys.

### 2.5. Data Evaluation

Data were evaluated using R programming software [21,22]. The non-parametric Mann–Whitney U test was chosen for the determination of statistical differences between groups at *p* < 0.05. The correlation of observed parameters was determined by an evaluation of the correlation coefficient (Pearson’s R). A coefficient greater than 0.8 and *p* < 0.05 were considered significant. Because of the large differences between individual donors/patients, the graphs show normalized data for a clearer display of trends (decrease/increase), where the original value (before HSCT) is taken as 100% and subsequent values are displayed as a percentage decrease/increase of the initial value.

## 3. Results

### 3.1. Expression of Inhibitory KIRs and Their Correlation with Clinical Data

**Figure 2:**
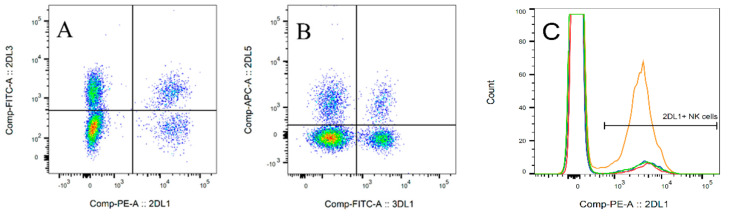
Representative dot-plots and histogram of inhibitory killer-immunoglobulin-like receptor (iKIR) evaluation by flow cytometry. The iKIRs were measured on the surface of NK cells, and the proportion of each iKIR was determined. The expression or coexpression of KIR2DL3 and KIR2DL1 (**A**) and KIR3DL1 and KIR2DL5 (**B**) is shown. The proportion of NK cells positive for KIR2DL1 changed over time (**C**). Orange line = before hematopoietic stem cell transplantation (HSCT); green line = 1 month after HSCT; blue line = 2 months after HSCT; red line = 3 months after HSCT.

We determined the percentage of NK cells positive for iKIRs by flow cytometry. iKIR expression on the surface of NK cells differed between the donors and also on the surface of recipients’ NK cells after transplantation (see Table 2 for the median and ranges of all evaluated iKIRs). In some donors, we did not detect all iKIR receptors; 2DL5 was expressed in just 50% of donors with a low proportion of positive NK cells (under 10%). The biggest differences in expression among individuals were detected in 3DL2 and 3DL1 receptors, where the variability ranged between 0% and 50% of positive NK cells. An example of the population distribution is shown in Figure 2 and Supplementary Materials, Figure S1, and the median and range of the positive cells at each time point are presented in Table 2.

**Figure 3:**
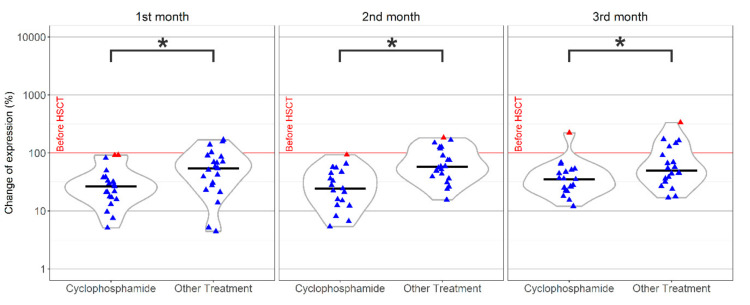
The percentage change of KIR2DL1 expression. The KIR2DL1 was downregulated in almost all patients and displayed different behaviors, depending on the graft-versus-host disease (GvHD) prevention treatment. The group receiving cyclophosphamide (Cy) exhibited a much more reduced expression than the group not receiving Cy. * statistically significant results (*p* < 0.05). Outliers, 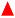. Median, 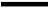.

The changes in iKIR expression at 1, 2, and 3 months post-HSCT normalized to donor values were correlated with clinical data, including diagnosis, CMV reactivation, the presence of relapse, the type of transplantation (mismatch, match), and the outcome of treatment (survival). The proportion of all iKIRs was significantly changed when the percentage of positive NK cells before and at the first and second month after HSCT was compared (*p* < 0.05). For the third month, only differences in KIR2DL1 and KIR2DL2 were statistically significant (also see Table 2). The most significant change was detected in the KIR2DL1 receptor. KIR2DL1 decreased in almost all patients (more than 85% of patients at all time points); in 50%, the expression was reduced by more than 50% of the original value (before HSCT) (Figure 3). The strongest downregulation was observed in patients receiving cyclophosphamide (Cy) in their GvHD prevention treatment (HLA mismatch transplantations), where the level was down to around 25% of the original value (Figure 3).

**Figure 4:**
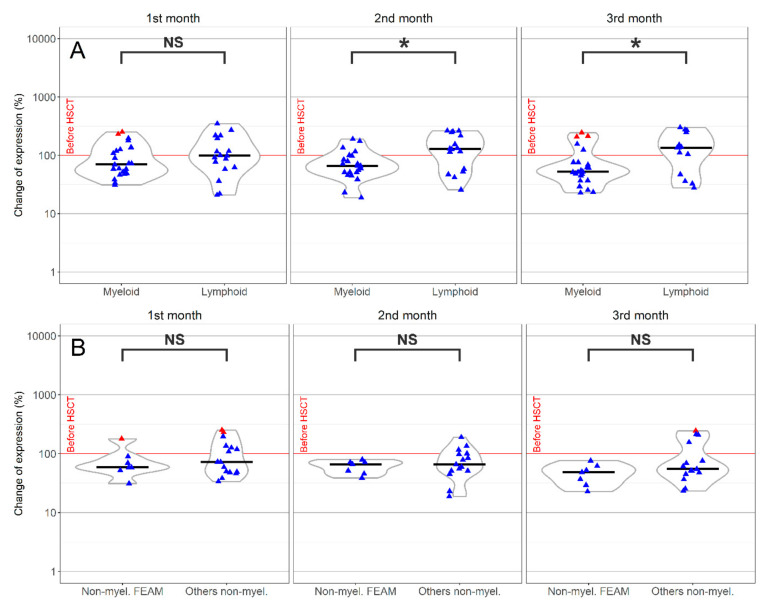
KIR2DL3 differential expression changes in the group of patients with myeloid neoplasia, where the expression decreased compared to the original value, and in a lymphoid group, with a higher expression of NK cells in patients than in the donor before HSCT (**A**). The FEAM (Fludarabine + Etoposid + Ara-C + Melphalan) protocol reduced the expression of KIR2DL3, unlike other conditioning regimens in the myeloid group (**B**). * statistically significant results *p* < 0.05; NS, without statistical significance (*p* > 0.05); outliers, 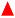; Median, 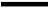.

Considering the underlying diagnosis of the recipient, the most striking effects were found in KIR2DL3, where we detected obvious differences among myeloid and lymphoid neoplasms (in the second and third month after HSCT *p* < 0.05). The KIR2DL3 expression was reduced with a time-dependent trend in more than 70% samples of the myeloid group (Figure 4A). In comparison, patients with lymphoid malignancy showed an increased expression in 80% of samples at the same time points (Figure 4A). This receptor was also affected by the disease status, requiring a different type of treatment/conditioning in myeloid malignancies. The decline of KIR2DL3 in patients, where FEAM (Fludarabine + Etoposid + Ara-C + Melphalan) was used, appeared to be more pronounced, but did not reach statistical significance (*p* > 0.05) (see Figure 4B).

**Figure 5:**
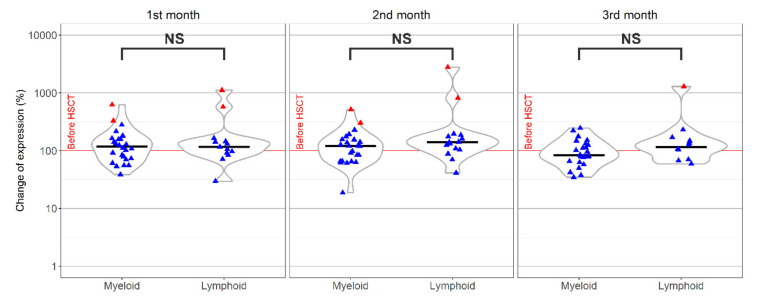
KIR3DL1 expression showed minimal changes, but slight differences (*p* = 0.06, on the border of statistical significance) were detected between myeloid malignancies and the lymphoid group. NS, without statistical significance; outliers, 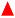; Median, 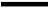.

KIR3DL1 expression exhibited different behaviors compared to previous ones. In lymphoid malignancies, the slight increase was stable in 75% of patients at all time points (Figure 5). In the myeloid group, however, the expression increased in over 50% of samples (the first month), and then started to decrease slightly in the second month (reduced in 50% of patients). Finally, in the third month, 68% showed a lower expression (Figure 5). The differences were not statistically significant. The behavior of other inhibitory KIRs (KIR2DL2 and KIR3DL2) was very heterogeneous. Other clinical parameters, such as the presence of CMV infection, early relapse (until 3 months after HSCT), the type of transplantation (mismatch, match), and the survival, were followed and correlated with the expression level of iKIRs, but there was no significant (*p* > 0.05) association.

### 3.2. Changes of KIRs During Cell Culture

**Figure 6:**
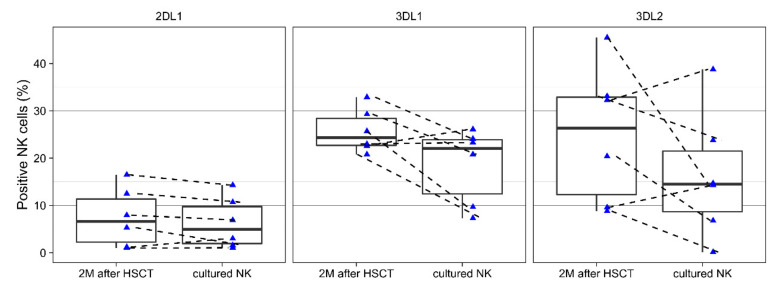
The expression of KIRs before (pink) and after (blue) ex vivo activation (n = 6). NK cells were isolated after HSCT and cultured for 18 days. The expression of iKIRs was estimated by flow cytometry. The cellular activation procedure did not cause significant changes in iKIR expression. No differences were statistically significant.

To evaluate the effect of ex vivo activation on iKIR expression, we cultured NK cells from patients 2 months after HSCT in the presence of allogeneic feeder cells (pooled MNCs from five donors) and IL-2 for 18 days. The purity of cultured cells was always above 90%; the cellular proliferation was comparable for all donors. The expression of all iKIRs changed after a culture period. In most cases, there was downregulation, with some exceptions, where the expression increased, but was not more than about 10% (Figure 6). Donors with no expression at the time of isolation maintained an undetectable expression after ex vivo activation.

The receptor that showed the most pronounced changes was KIR2DL1, with only 10% of patients exhibiting an increase. Therefore, we decided to test its importance in the anti-tumor response. We tested NK cells’ cytotoxic potential against NK-resistant AML (acute myeloid leukemia) cell line KG1a. To block the KIR2DL1–HLA-C interaction, we cultured NK cells in the presence of blocking anti-KIR2DL1 antibody for 2 h and subsequently added KG1a at a 10:1 ratio (NK:KG1a) for 24 h. Using the AML specific marker CD34, we measured the proportion of dead CD34 positive cells using 7AAD dye for dead cell identification.

NK cells showed low cytotoxic activity against KG1a cells (around 20–30% after 24 h co-culture). Inhibition of the KIR–HLA interaction did not improve the killing ability of NK cells, and KG1a cells could still resist the NK killing (see Figure S2A–C, Table S1).

Next-generation sequencing was used for the genotyping of all donors. We identified several alleles in all genes, with the highest heterogeneity in *KIR3DL2*, where two times more combinations were detected. The most frequent allele for *KIR2DL1* was *002 (more than 1/3 of donors); for *KIR2DL2*, was *003 (1/2); for *KIR2DL3*, was *001 (1/2); for *KIR3DL1*, was *001 (1/3); and for *KIR3DL2*, was *001. The presence of *KIR2DL5* was detected in about 2/3 of patients (with the dominance of KIR2DL5A*005-KIR2DL5B*002), but some of them were not transcribed. We tested the influence of KIR gene polymorphism and the surface expression of the corresponding protein, but we did not detect any association between the presence of allele and protein expression.

## 4. Discussion

Allogeneic hematopoietic stem cell transplantation (HSCT) is an effective therapy for many hematological malignancies; however, relapse still represents an essential cause of treatment failure. NK cells are one of the critical players in anti-tumor immunity and are considered crucial immune cells in early relapse control. Using autologous NK cells from allogeneic transplanted patients has great potential. However, there are still many unanswered questions, such as those relating to the time of cell collection or the composition of receptors and the level of their expression. KIRs are a family of transmembrane glycoproteins regulating the development, activation, and tolerance of NK cells [23]. KIR receptors are highly polymorphic, and polymorphism generates a wide variety of haplotypes that differ among individuals and populations [24], where a spectrum of common alleles and rare alleles can be found [25,26]. KIR expression after HSCT is a dynamic process with vast differences within individuals. A previous study following the mRNA level of iKIRs after HSCT detected changes in the mRNA level in the second and third months after HSCT, when the mRNA expression was compared for donors and patients after HSTC [27]. In the study, the mRNA level of KIR2DL1 was higher in transplanted NK cells, and it is opposite to what our study shows, where the surface expression of this receptor was significantly decreased in almost all patients, in concordance with other similar studies [28,29]. mRNA levels from the heterogenic population may not reflect the surface expression on NK cells. The expression of KIR can also be regulated post-transcriptionally [30]. Therefore, immunophenotyping is the only valid method for studying functional KIR behavior in the NK population.

NK cells do not express the full spectrum of KIR receptors on their surface, and the level of KIRs is affected by several factors, such as virus infection (CMV) [31] or autoimmune disease [32]. A correlation between KIR2DL5 and CMV was observed in renal transplantation, where patients carrying the *KIR2DL5* gene were more prone to CMV infection [33], but was not clearly proven in HSCT, where the virus infection seems to be controlled by the whole spectrum of KIRs and the level of interaction of KIR and their ligands [34]. In addition, the proportion of NK cells expressing KIR2DL5 is very low and usually under 10% [35]; in our study, only 50% of donors showed a proportion of KIR2DL5 positive NK cells higher than 0.5%. The expression of other iKIRs is usually higher, but even so, there is great heterogeneity between individuals.

In the presented study, we saw significant changes in several iKIR expressions. A noticeable reduction was observed in KIR2DL1, especially in the HLA mismatch transplantation, where Cy is used as GvHD prophylaxis. In these patients, the level of KIR2DL1 was only around 25% of the original level. A downregulation of KIR2DL1 gene expression by Cy has been shown within 6 h after receiving Cy treatment [36]. Our research shows that the long-term effect of Cy on KIR2DL1 expression can last at least 3 months. A dynamic expression was also detected in other inhibitory KIRs. The nature of the malignancy can influence the differential expression of some iKIRs; the KIR2DL3 showed different behavior in a myeloid group, with lower frequencies of positive cells compared to lymfoid patients, where the frequencies were the same or even higher than on the cells before transplantation. This receptor is the first expressed on recovered NK cells after HSCT [37], and its level might be associated with the conditioning treatment or disease persistence. The decrease was more evident in patients using FEAM for the conditioning, which is mostly employed in patients with active disease before HSCT. Therefore, we cannot exclude the influence of residual blasts and their interaction with NK cells, but in the case of KIR2DL3, the expression in patients with myeloid leukemia is usually rather elevated [38], suggesting the effect of the used chemotherapy, rather than the remaining blasts, on the level of KIR2DL3. Interestingly, we also saw different behaviors of KIR3DL1 expression in myeloid and lymphoid leukemias, but there was no leukemic blast infiltration during the first three months, suggesting the influence of pre-transplant treatment, rather than the type of leukemia.

KIR–HLA interaction is studied mostly in the context of relapse control and improving NK cells’ response. In the clinic, KIR–HLA interaction is blocked using the antibody Lirilumab, which binds KIR2DL1, -2, and -3 receptors [39]. Despite its positive influence on the HSCT outcome, there is still the potential for NK cell blocking through KIR3DL receptors. We tested the blocking of the KIR2DL1 receptor (with low expression in 90% of patients), but we did not see any improvement in the NK killing ability when only KIR2DL1 was blocked. This is in concordance with our previous study showing the greater importance of increasing the activation signal than decreasing the inhibitory one [40]. Moreover, the activation of NK cells did not significantly increase the expression of inhibitory KIRs, but significantly upregulated activating receptors such as NKG2D. This opens the possibility of using NK cells regenerated in patients after HSCT and allows the use of ex vivo activated cells in all patients with high-risk disease or relapse enrolled in clinical trials, eliminating the need for healthy donors for NK cell donation. Therefore, for the purpose of NK cell therapy, the optimal time of cell collection is defined by the sufficient number of NK cells, rather than iKIR expression. The clinical protocol based on our above findings is already being used in the ongoing clinical trial EudraCT number: 2018-001562-42, designed for the prevention and treatment of post-transplant relapses with cytotoxic highly activated NK cells.

## 5. Conclusions

Our initial study confirms the dynamic surface expression of inhibitory KIRs after HSCT and its correlation with clinical data, such as diagnosis and the conditioning protocol. We determined the important role of KIR expression monitoring for the appropriate evaluation of the KIR–HLA interaction and the need to measure the surface expression instead of the genotype or mRNA only. A future more extensive multi-centric study measuring the KIR expression in transplanted patients will allow a deeper understanding of the influence of clinical parameters on the expression of KIR receptors and can also address the association of the expression with the patient’s outcome.

## Figures and Tables

**Table 1:** Clinical structure of the study cohort.

Characteristic	Type	No. of Individuals		
Diagnosis	Myeloid	27		
	Lymphoid	17		
Sex (patients)	Male	15		
	Female	29		
Age (patients)			Median: 50	Range: 20–74
Sex (donors)	Male	28		
	Female	16		
Age (donors)			Median: 37.5	Range: 20–60
HSCT type	Matched	19		
	Mismatched	25		
Disease status	CR	25		
(at time of HSCT)	Non CR	19		
Conditioning	Myeloablative	5		
	Non-myeloablative	39		
Post-transplant treatment	Cyclophosphamide	22		
	Others	22		

**Table 2:** Percentage of iKIR positive NK cells (%). All iKIRs were different when the percentages of positive NK cells before HSCT and 1 and 2 months after HSCT were compared. Statistically significant results (*p* < 0.05) are denoted by *.

Time Point		3DL1	3DL2+/3DL1−	3DL2+/3DL1+	2DL5	2DL3	2DL1	2DL2+/2DL3+	2DL2+/2DL3−	2DL1+/2DL3+
Before HSCT	Median	17.1	1.6	21.5	0.2	15.8	16.6	33.8	14.4	3.7
	(Range)	(0–47.9)	(0–23.7)	(0.2–50.8)	(0–4.8)	(0.2–74.2)	(0.2–46)	(16.2–76)	(1.9–50.2)	(0–35)
1 month *	Median	18.5	4.2	24.2	0.2	17.5	5.0	27.2	7.1	2.1
	(Range)	(0–46.9)	(0.5–63.5)	(0.7–77.1)	(0–9.3)	(0–40.6)	(0.4–37.9)	(3.9–58.1)	(0.6–49.5)	(0–15)
2 months *	Median	20.5	2.7	25.4	0.2	17.5	5.8	27.0	6.6	2.7
	(Range)	(0–46)	(0.1–51.2)	(0.2–74.2)	(0–6.6)	(0.1–36.9)	(0.1–40.7)	(7.1–52.6)	(0.2–41.3)	(0–16.6)
3 months	Median	18.0	2.3	20.7	0.2	16.7	7.8 *	25.0 *	9.9	2.7
	(Range)	(0–60)	(0–24.7)	(0.5–62.5)	(0–10.9)	(0.6–37.5)	(1.2–40.4)	(10.4–58)	(0.5–38.9)	(0.2–21.6)

## Supplementary Materials

The following are available online at https://www.mdpi.com/2077-0383/9/11/3502/s1; Figure S1: The KIR receptor evaluation gating strategy; Figure S2: Determination of the cytotoxic activity of NK cells.; Table S1: The role of KIR2DL1 blocking.
