# Supplementary material for: Dynamic Changes of Inhibitory Killer-Immunoglobulin-Like Receptors on NK Cells after Allogeneic Hematopoietic Stem Cell Transplantation: An Initial Study
Source: J Clin Med. 2020 Oct 29;9(11):3502. doi: 10.3390/jcm9113502 (PMC7692795; doi:10.3390/jcm9113502)
Supplement: Supplementary file 1 [file jcm-09-03502-s001.pdf]

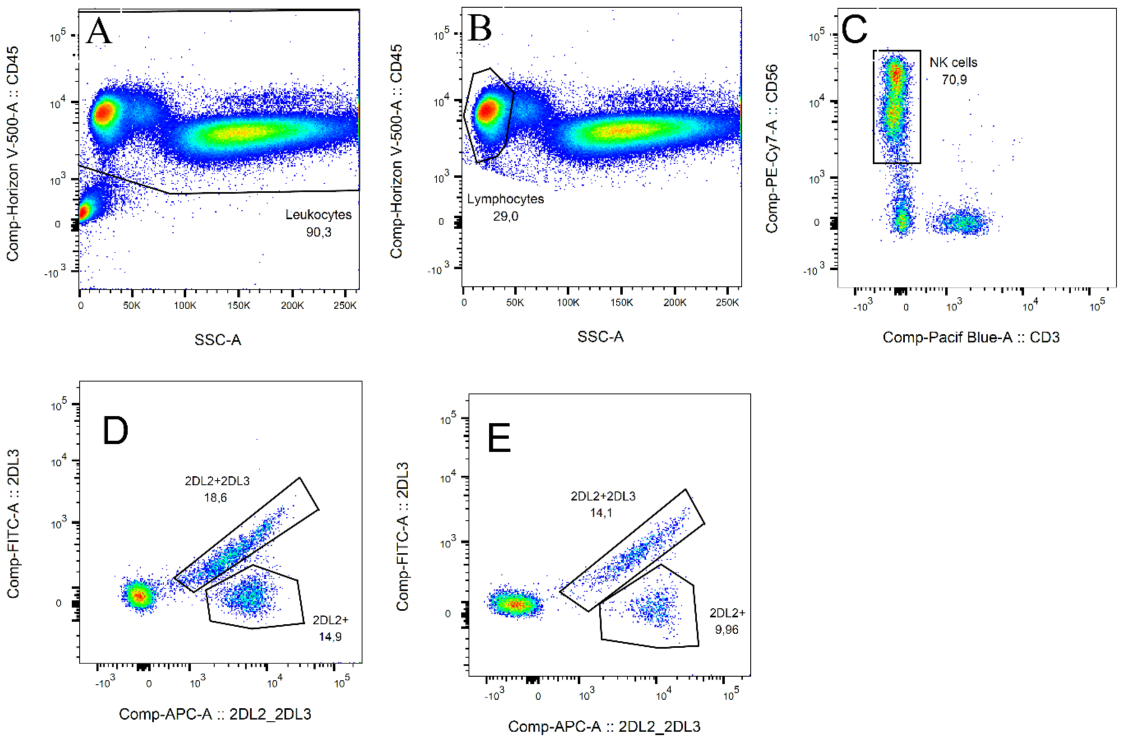

Figure S1. The KIR receptor evaluation gating strategy. The leukocytes were chosen based on CD45 expression (A) following by the detection of lymphocytes with low SSC (B). NK cells were determined using CD56 positivity and CD3 negativity (C). KIRs were then gated from NK cells. The difference proportion of KIR2DL2 cells was observed when sample before HSCT (D) and 2 months after HSCT (E) was compared.

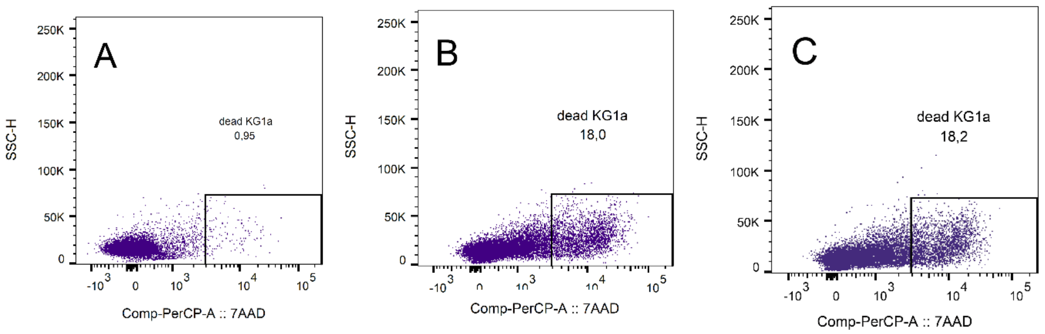

Figure S2. Determination of the cytotoxic activity of NK cells. Cytotoxic activity against NK-cells resistant cell line KG1a was determined in co-cultured experiments where KIR2DL1-HLA was blocked by an antibody against KIR2DL1. The proportion of dead KG1a cells in culture without NK cells (A), with NK cells together with anti-KIR2DL1 antibody (B), with NK cells only (C) was estimated. No difference was detected (n=4).

Table S1. The role of KIR2DL1 blocking. Proportion of dead cells in cell culture with NK cells combining with anti-KIR2DKL1 blocking NK cells only.

Identification of NK cells	NK and 2DL1 blocking combination	NK cells only
D1	19.95 (±1.65)	20.05 (±1.25)
D2	28 (±0.9)	27.25 (±0.95)
D3	19.15 (±0.95)	19.05 (±1.05)
D4	23.35 (±0.35)	20.05 (±0.55)
